# Hydrodynamics of Vortex Generation during Bell Contraction by the Hydromedusa *Eutonina indicans* (Romanes, 1876)

**DOI:** 10.3390/biomimetics4030044

**Published:** 2019-07-05

**Authors:** John H. Costello, Sean P. Colin, Brad J. Gemmell, John O. Dabiri

**Affiliations:** 1 Biology Department, Providence College, Providence, RI 02918, USA; 2 Whitman Center, Marine Biological Laboratory, Woods Hole, MA 02543, USA; 3 Marine Biology/Environmental Sciences, Roger Williams University, Bristol, RI 02809, USA; 4 Department of Integrative Biology, University of South Florida, Tampa, FL 66320, USA; 5 School of Engineering, Stanford University, Stanford, CA 94306, USA

**Keywords:** swimming, vortex rings, wakes

## Abstract

Swimming bell kinematics and hydrodynamic wake structures were documented during multiple pulsation cycles of a *Eutonina indicans* (Romanes, 1876) medusa swimming in a predominantly linear path. Bell contractions produced pairs of vortex rings with opposite rotational sense. Analyses of the momentum flux in these wake structures demonstrated that vortex dynamics related directly to variations in the medusa swimming speed. Furthermore, a bulk of the momentum flux in the wake was concentrated spatially at the interfaces between oppositely rotating vortices rings. Similar thrust-producing wake structures have been described in models of fish swimming, which posit vortex rings as vehicles for energy transport from locations of body bending to regions where interacting pairs of opposite-sign vortex rings accelerate the flow into linear propulsive jets. These findings support efforts toward soft robotic biomimetic propulsion.

## 1. Introduction

Medusae present an important opportunity for understanding animal propulsion because they were likely the earliest animal phyla to develop muscular swimming. Cnidarians are one of the most ancient animal lineages [1,2] and medusan fossils very similar in morphology to extant medusan clades have been described from marine environments as old as 505 million years ago [3]—probably before the evolution of vertebrate fish or other animal groups invaded terrestrial and aerial environments. A fundamental limitation of medusan muscle arrays is that cnidarian muscle fibers are housed solely within epitheliomuscular cells. The single-cell thick nature of epithelial cells limits the thickness of swimming muscle arrays within cnidarians and, consequently, force production during medusan swimming. As a result, effective medusan propulsion must operate within phylogenetically-constrained limits on force production. The very basic level of their swimming machinery points to a question that may be insightful for understanding both biological and biologically-inspired propulsive systems—how can a set of comparatively simple biological components be organized into such a successful propulsive system?

The early focus of medusan swimming mechanics centered on jet-propelled species. Early descriptions of jet propulsion depended upon contraction of muscular fibers encircling the swimming bell to eject fluid that is accelerated into a thrust-generating jet as it passes through a narrow aperture, the velum. A model of this process [4], involving the change in swimming bell volume and velar aperture width during bell contraction, yielded kinematic estimates of swimming motion that were consistent with observed jet-propelled medusan swimming [5]. Further examination of medusan swimming expanded the range of propulsive modes used by medusae—beyond simple jetting—to include the use of flexible bell margins by oblate species in a type of rowing motion [6,7,8,9]. These studies, in common with research on other animal swimmers (e.g., [10,11]) and fliers (e.g., [12,13]) have identified energy transfer through vortex ring formation within animal wakes as an essential process for understanding animal propulsion in inertial fluid regimes. Although essential traits of wake structure have been clarified for medusae [14], the generation of vortex rings and their interactions during thrust production have remained only vaguely understood. A clearer empirical understanding of these interactions would benefit animal biology and is essential for replicating animal propulsion by engineered vehicles [15].

One obstacle to documenting flows of swimming medusae is that flow initiation occurs within the subumbrellar cavity, a region often surrounded by body tissue with limited transparency. Measurements of these flows are hindered when the medusa’s body wall obscures the ability to observe flow patterns. This limitation can be at least partially overcome by using target species possessing a highly transparent body that permits imaging through the body wall and into the enclosed subumbrellar fluids. The hydromedusa *Eutonina indicans* is such a species which, in combination with digital particle imaging velocimetry methods [14,15], provides an opportunity to quantify the fluid interactions underlying propulsion by medusae. This new knowledge can be used to inspire biomimetic propulsion by means of soft robotic actuation techniques that have recently been developed [8].

*Eutonina indicans* is a jetting medusa. However, unlike some other hydromedusae, it does not possess a pronounced velum at the oral end of its body. This reduced velar component facilitates the aforementioned optical access to the flow in the subumbrellar cavity. Moreover, the oblate fineness ratio (i.e., the height to diameter ratio) of its body relative to other jetters made it a compelling scientific focus for this study, as its body plan is intermediate between many jetters and rowers [14]. Thus, the fluid mechanics revealed in this study have the potential to inform biomimetic designs that aim to replicate both classes of medusae. 

## 2. Materials and Methods

*Eutonina indicans* medusae (0.7–1.3 cm relaxed bell diameter) were obtained from the New England Aquarium and maintained in 20 L aquaria at 20 C. Swimming kinematics and fluid interactions during swimming were measured for individuals placed into a glass filming vessel seeded with neutrally buoyant, hollow glass spheres (10 μm). Only recordings of animals swimming upwards were used in the analysis to eliminate the possibly of gravitational force from aiding forward motion of the animal between pulses. One swimming sequence with multiple pulsation cycles (n = 5) was selected for detailed study of kinematic and fluid dynamic data.

Medusae were illuminated using a 680-nm wavelength laser sheet and recorded at 1000 frames s^−1^ using a high-speed digital video camera (Fastcam 1024PCI; Photron, San Diego, CA, USA) placed perpendicular to the laser sheet. The laser sheet illuminated a two-dimensional sheet of the glass spheres around the medusae and the cross-section through the center of the medusan bell. Velocities of particles illuminated in the sheet were determined using digital particle image velocimetry (DPIV) software (Lavision Inc., Ypsilanti, MI, USA) that analyzed sequential video frames using a cross-correlation algorithm. Image pairs separated in time by 6 ms were analyzed with shifting, 50% overlapping interrogation windows of decreasing size (i.e., 32 × 32 pixels, followed by 16 × 16 pixels). Prior testing with these interrogation window settings and interval duration demonstrated >2 pixel average particle motion between image pairs, as well as the most consistent velocity and vorticity estimates at the experimental seeding density and magnification. This analysis generated a 128 × 128 gird of velocity vectors around the swimming medusae. 

Bell kinematics, such as subumbrellar volume and velar aperture diameter, were quantified from the cross-sectional images of the bell and were analyzed using Image J software (NIH, Bethesda, MD, USA). The bell and swimming kinematics were quantified using previously reported methods [5]. Changes in bell shape were quantified as the fineness ratio, *f*, where

(1) $$f=\frac{\mathrm{bell}\;\mathrm{height}}{\mathrm{bell}\;\mathrm{diameter}}$$

and swimming speed, *U*, as:

(2) $$U\;=\;\frac{{\left({\left(x_{2}-x_{1}\right)}^{2}+{\left(y_{2}-y_{1}\right)}^{2}\right)}^{1/2}}{t_{2}-t_{1}}$$

where *x* and *y* are the coordinates of the apex of the bell in successive images. Pixel discretization errors were filtered by neglecting bell position changes of less than 33 μm. This was <20% of average position changes occurring during periods of bell contraction and forward swimming.

Analyses of wake structures were performed from DPIV velocity vector fields. The contribution of different fluid regions to the starting vortex rings during bell contraction was measured as the momentum flux (*p_flux_*) of fluid across transects positioned across the velar diameter at the fluid exit from the subumbrellar cavity. The video frames were re-oriented so that the trajectory of swimming medusae was parallel to the y-axis (i.e., vertical). With this orientation, the velocity of the jet of fluid emerging from the subumbrellar cavity had a predominant y-component (*u_y_*). The momentum flux [15,16] was estimated for a linear transect across the jet emerging from the velar aperture (*p_flux_*) using the fluid velocity vector (**u**), the direction perpendicular to the linear transection (**n**), and the y-component of the jet (*u_y_*):

(3) $$p_{flux}=\int {}_{exit}u_{y}(u\cdot n)dA\approx \frac{\pi \rho u_{y}^{2}l^{2}}{4}$$

where *ρ* is the density of seawater and *l* is the length of the horizontal transect. Estimation of total momentum flux through the velar aperture assumed a circular velar aperture area with a diameter of the transect line. 

Circulation (Γ) within starting vortex rings on either side of the bell margin was quantified as:

(4) Γ(t)=∫ω(x,y,t)dxdy

where *ω* is the out-of-plane vorticity of the fluid. The wake circulation is subsequently reported as the sum of the magnitude of vortex ring circulation on both sides of the bell margin, in order to account for measurement variability due to the laser sheet being potentially offset from the symmetry plane of the wake vortices. Dividing this total circulation magnitude by two gives the average vortex ring circulation.

## 3. Results

### 3.1. Body Kinematics and Vortex Development

**Figure 1:**
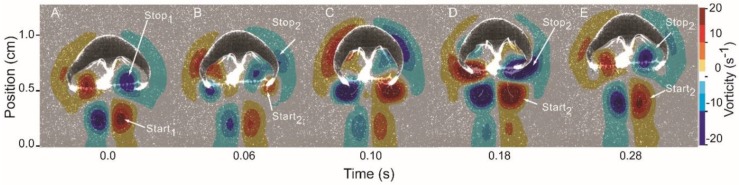
Vortex circulation development during bell pulsation by the hydromedusa *Eutonina indicans*. (**A**) Relaxed bell position between contraction cycles of a medusa (0.74 cm relaxed bell diameter) swimming in a linear, vertical direction. Residual circulation of the previous pulsation cycle is evident as starting vortex flow in the wake (Start_1_) and opposite-sign flow in the subumbrellar cavity (Stop_1_); (**B**) Initiation of bell contraction with starting vortex formation at the bell margin (Start_2_) accompanied by an increase in stopping vortex circulation in the mid-bell region (Stop_2_); (**C**) Continued bell contraction and increased circulation of both starting and stopping vortices; (**D**) Separation of starting vortex ring (symmetrical, opposite-sign on either side of the bell) from the bell margin with movement of the stopping vortex circulation along the exumbrellar surfaces past the bell margin and into the subumbrellar cavity during the refilling phase of bell relaxation; and (**E**) Return to the relaxed bell position between contraction cycles. The remnants of starting and stopping vortices remain in the wake and within the subumbrellar cavity, similarly to the end of the previous pulsation cycle (panel A).

Swimming *Eutonina indicans* medusae generated a characteristic series of opposite-sign vortex rings during bell pulsation. Starting from rest (Figure 1A), the bell contracted, driving the bell margins towards the central axis of the medusae. Movement of the bell margin towards the central axis was accompanied by formation of a starting vortex ring along the bell margin (Figure 1B). Simultaneously, a stopping vortex ring, characterized by rotational flow in the opposite direction to that of the starting vortex, was initiated near the middle of the bell, primarily along the mid-exumbrellar surface. When swimming in a linear direction, the formation of this starting–stopping pair of vortices was largely synchronous around the bell and resulted in symmetrical, opposite-sign circulation on either side of the bell, as documented by two-dimensional DPIV measurements. Both sides of the bell acted symmetrically during the bell pulsation cycles of linear swimming. As the bell contracted further, the starting vortex ring continued development along the bell margin and came into contact with residual stopping-vortex circulation fluid that was ejected from the subumbrellar cavity (Figure 1B,C). At the end of bell contraction, the starting vortices on either side of the bell were shed from the bell margin into the wake trailing the medusa (Figure 1D). By that time, circulation from the next stopping vortex, which initially formed near the mid-bell regions during bell contraction, had moved down the bell to its margin. During bell relaxation, this vorticity from the stopping vortex advected around the bell margin and into the bell cavity, refilling the subumbrellar cavity. This fluid frequently continued to rotate within the bell cavity after the bell was refilled (Figure 1A,E). Subsequent bell contraction forced the fluid possessing stopping vorticity out of the bell velum where it converged and interacted with the starting vortex of the new contraction cycle (Figure 1B).

**Figure 2:**
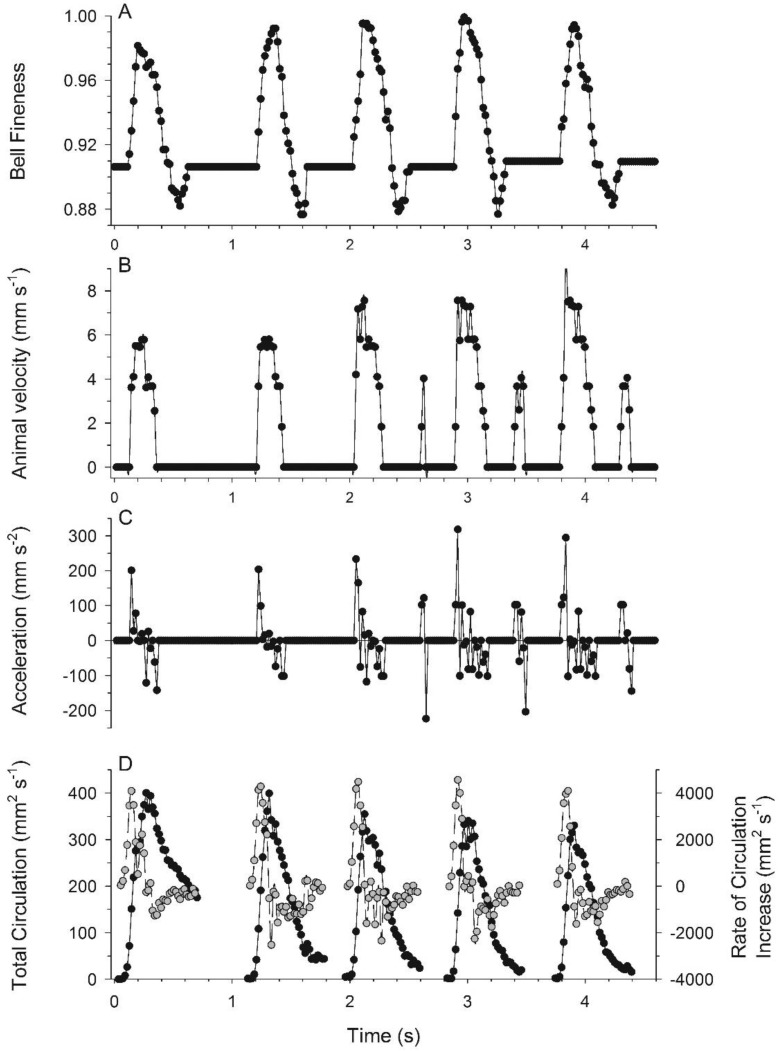
Swimming movements and vortex circulation patterns during linear swimming by the hydromedusa *Eutonina indicans*. (**A**) Bell fineness patterns over 5 consecutive pulsation cycles for a 1.01 cm bell diameter medusa; (**B**) body velocity; (**C**) acceleration; and (**D**) total starting vortex circulation (dark circles) and the rate of change in total starting vortex circulation (grey circles) during the same intervals.

**Figure 3:**
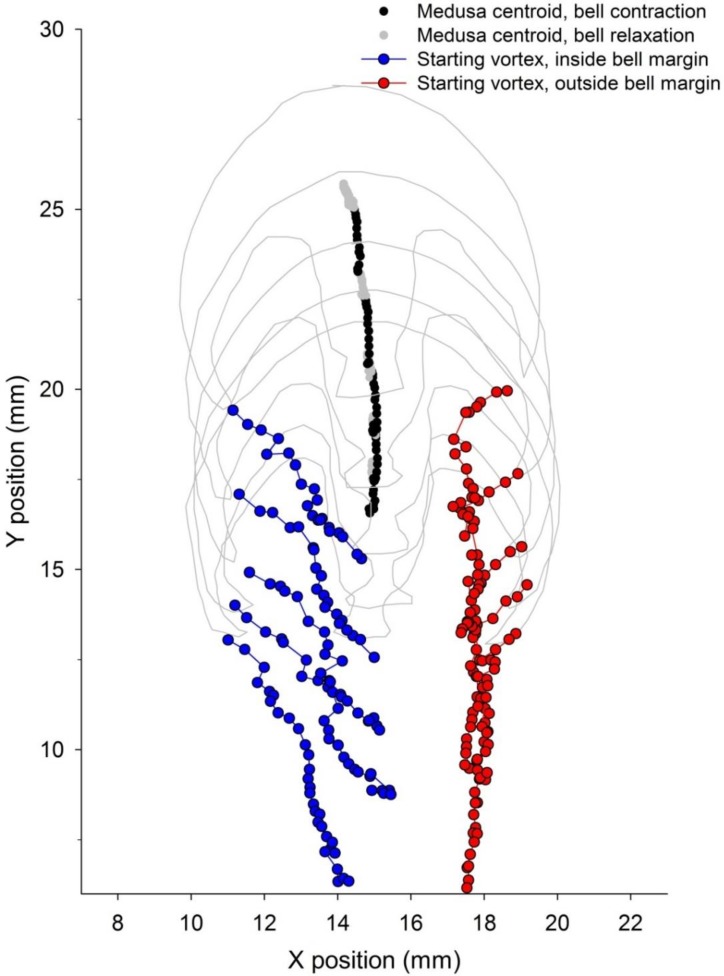
Paths of swimming *Eutonina indicans* medusa (same animal and sequence as depicted in Figure 2) and starting vortices generated in its wake. Animal outlines represent the body shape and position at the end of consecutive pulsation cycles. Centroids of the medusa body during pulsation (black—contraction phase, grey—relaxation phase) and starting vortices (red, blue) indicate the relative positions of the animal and the vortices it left in its wake during swimming. In this case, the animal’s path veered to the left during swimming and vortex pathways varied slightly from the inside (left—blue) to the outside (right—red) margins during the swimming sequence.

Movement of medusae through water occurred in a cyclic pattern matching vortex circulation production. Bell contraction, measured as elevated bell fineness (Figure 2A), was accompanied by rapid acceleration to peak velocities (Figure 2B,C). Starting vortex circulation peaked during bell contraction (Figure 2D) and periods of greatest rate of circulation increase corresponded with peak animal acceleration during each pulsation cycle (Figure 2C,D). Although overall animal position changes were relatively linear for the individual examined in detail, starting vortex pathways varied slightly on either side of the bell. The medusa’s path veered slightly and the starting vortex at the bell margin to the inside of the turn was directed more towards the central axis of the medusae than were the starting vortex centroids formed at the outside margin of the turn (Figure 3).

The relatively rapid return to zero swimming velocity (Figure 2B) likely reflects the lower swimming Reynolds number (<10^2^) of *Eutonina* relative to prior studies [9], which indicate a more pronounced coasting phase at higher Reynolds numbers. Nonetheless, passive energy recovery is still apparent in a transient coasting phase increase in velocity in the last three bell contractions.

### 3.2. Vortex Interactions, Momentum Transfer, and Body Motion

**Figure 4:**
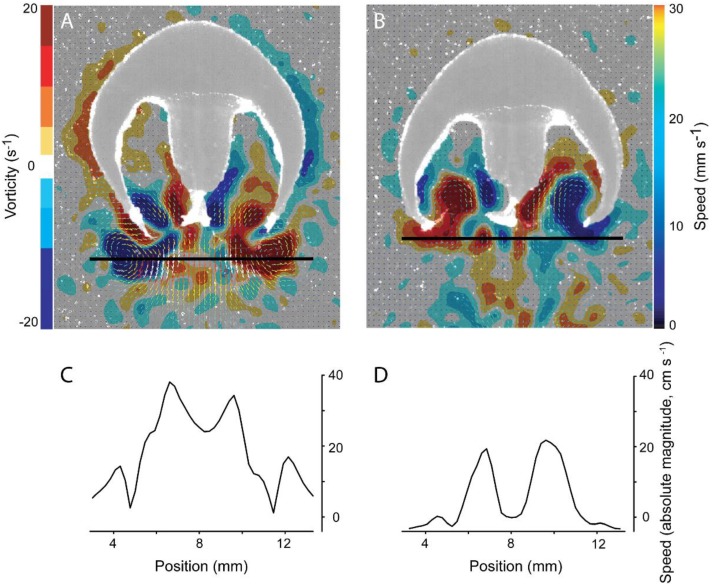
Vortex interface acceleration (VIA) during bell contraction and relaxation of *Eutonina indicans*. Top images were taken during (**A**) bell contraction and (**B**) bell relaxation of the second pulsation cycle in Figure 2. White vector arrows represent vector magnitudes above the scale limit. Lower panel images (**C**,**D**) indicate absolute values of vector speeds along transects corresponding to black transect lines (A,B). Note that flows are primarily outward from the bell during contraction and into the bell subumbrellar cavity during relaxation. Peak vector magnitudes occur at interfaces of fluid parcels having opposite sense circulation for both bell contraction and relaxation.

**Figure 5:**
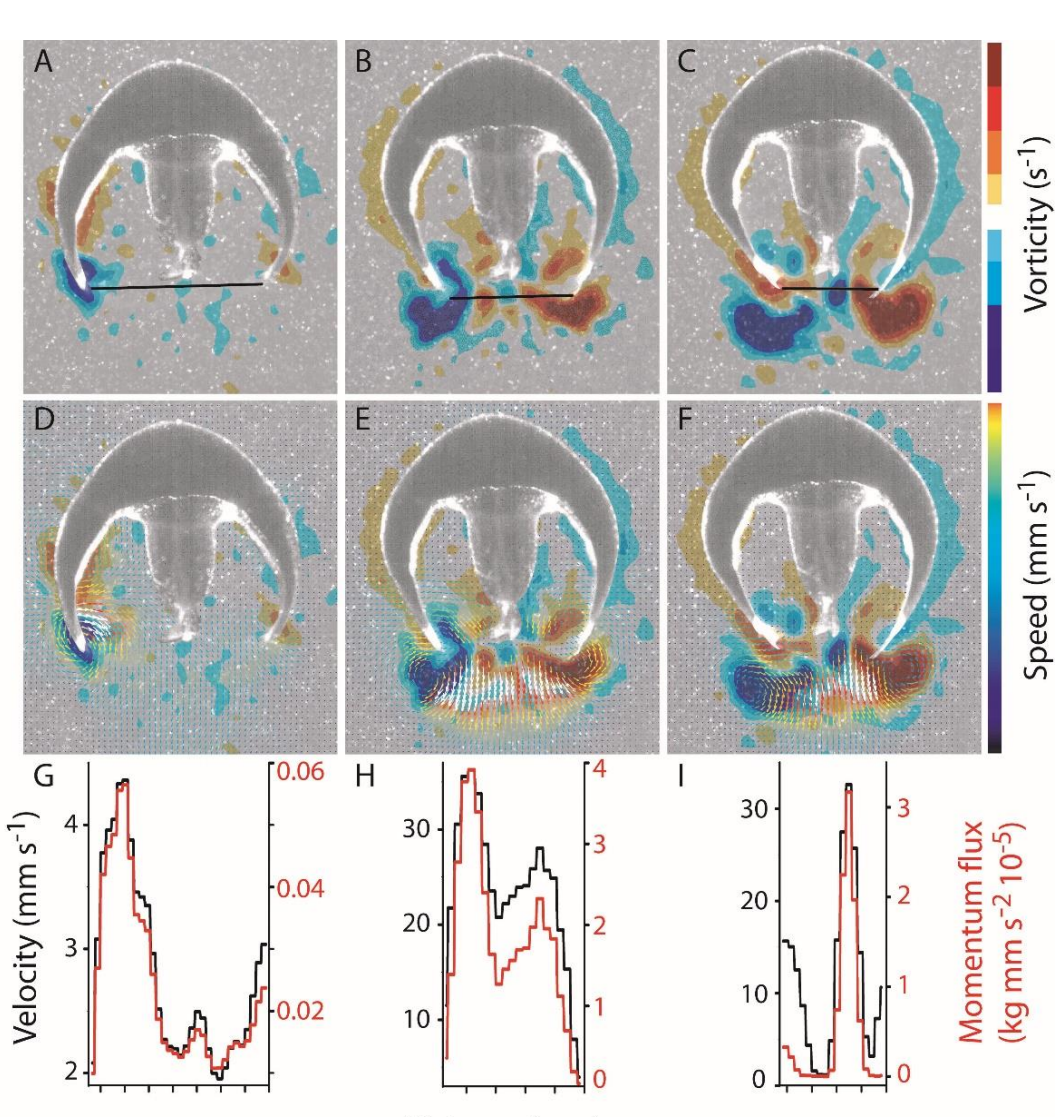
Body motions and vortex interactions during bell contraction by the hydromedusa *Eutonina indicans.* All data and animal images correspond to stages within the second pulsation cycle of Figure 2 and Figure 3. Columns represent different stages of pulsation: Peak acceleration at initiation of bell contraction (**A**,**D**,**G**); peak change in combined starting vortex circulation (**B**,**E**,**H**); and peak change in maximum vector speeds of combined VIA jets (**C**,**F**,**I**). Columns represent different stages of pulsation: Rows of images represent different parameters for each of these pulsation stages: Vorticity (**A**–**C**); vorticity and vector speeds (**D**–**F**); and velocity and momentum flux across transects (straight black lines in panels (**A**–**C**)) at the edge of the bell margins. The absolute values of vorticity and vector speeds varied throughout the pulsation cycle and, therefore, absolute magnitudes are not listed for the color scales of vorticity and vector speed. Note that momentum flux away from the animal corresponds to maximum flows at VIA regions of the wake and that VIA regions shift locations due to vortex movements during bell contraction. Momentum flux magnitude in (**G**–**I**) is given for each transect segment, as indicated by the discretization of the curves.

Vortex interactions dominated momentum transfer from the swimming medusa to the fluid in the medusa’s wake. Bell contraction forced fluid with stopping vortex circulation out of the bell cavity and through the expanded velar aperture. As this fluid was ejected, it was forced into contact with fluid characterized by opposite-sense rotation that was part of the developing starting vortex formed at the bell margin (Figure 1B and Figure 4A). The two fluid parcels of opposite-sense rotation did not mix where they met; instead, each fluid parcel maintained its rotational identity and, together, they formed an interface where fluid was accelerated to high velocities relative to the flows within the vortices on either side of the interface (Figure 4). Maximum vector speeds at these interfaces approached an order of magnitude higher than speeds on the opposite side of the vortex core, away from the stopping–starting vortex interface. These regions of vortex interface acceleration (VIA), which persisted until the stopping vortex translated away from the starting vortex into the subumbrellar region, were the dominant locations of momentum flux during the bell contraction cycle (Figure 5).

**Figure 6:**
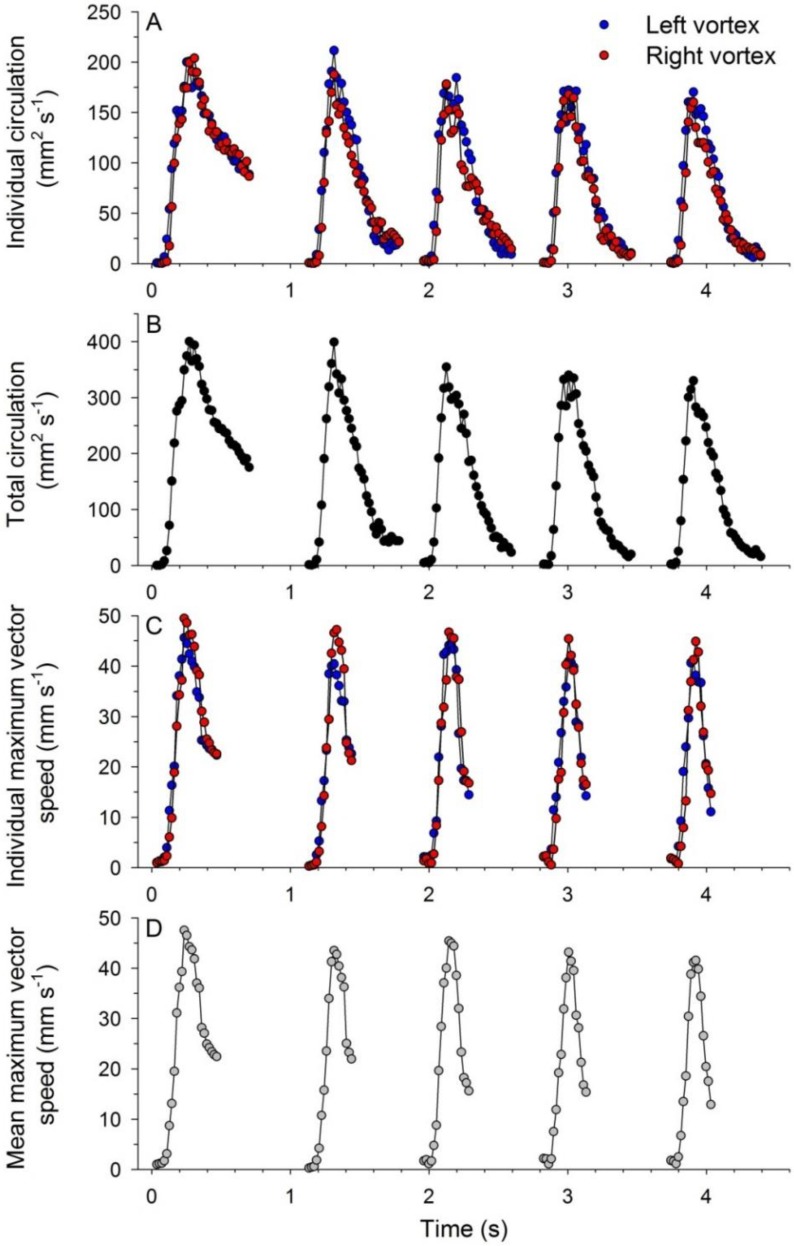
Vortex circulation and VIA magnitudes of starting vortices for consecutive bell pulsation cycles of a *Eutonina indicans* medusa swimming in a primarily linear direction. All data and animal images correspond to the pulsation cycles depicted in Figure 2 and Figure 3. (**A**) Starting vortex circulation magnitude at either bell margin; (**B**) combined total circulation for the two margins; (**C**) maximum vector speeds along VIA transects for either side of the bell margin; and (**D**) mean values for the combined maximum VIA region velocities.

**Figure 7:**
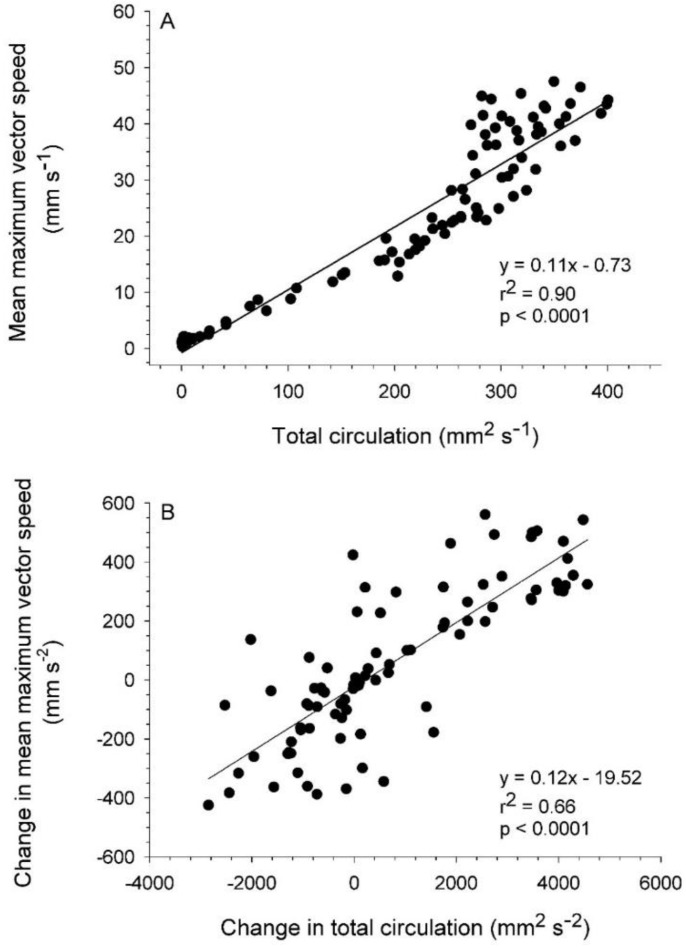
Linear correspondence between starting vortex circulation magnitude and VIA jet magnitudes for the hydromedusa *Eutonina indicans*. (**A**) Total, or combined, circulation of starting vortices compared to the mean of maximum VIA vector speeds adjoining both vortices and (**B**) change in total circulation and mean maximum vector speeds. All data represent 2D measurements derived from particle image velocimetry (PIV) images for the pulsation cycles depicted in Figure 2 and Figure 3.

**Figure 8:**
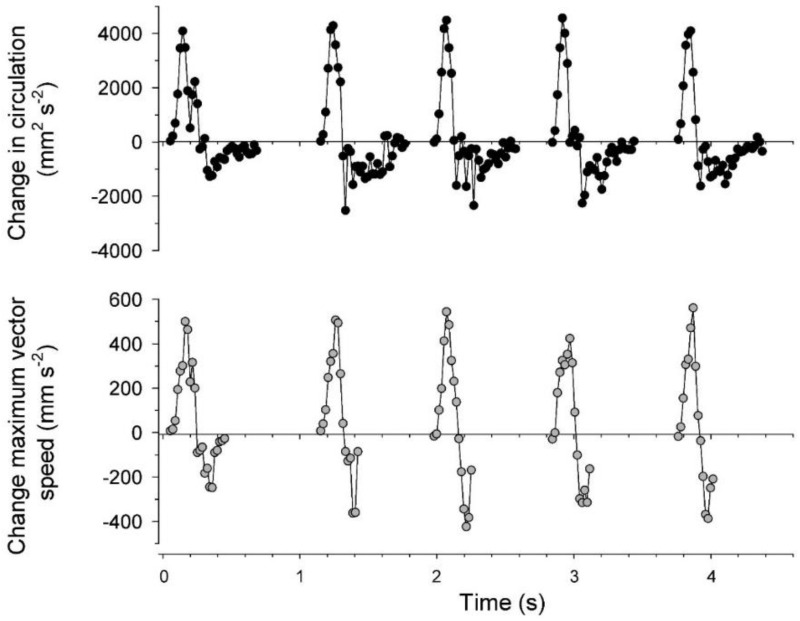
Correspondence between the change in combined circulation of starting vortices and the VIA region fluid speeds during bell contraction. All data represent 2D measurements derived from PIV images for the pulsation cycles depicted in Figure 2 and Figure 3.

The velocity magnitudes at VIA regions, and consequently momentum flux patterns, were directly related to circulation magnitudes of the interacting vortices. Starting vortex ring circulation magnitudes were approximately equal on either side of the bell (Figure 6) and maximum VIA region velocities were significantly linearly related to total starting vortex circulation magnitudes (Figure 7). Likewise, the rate of change in both total starting vortex circulation and maximum VIA jet speeds corresponded closely throughout bell contraction cycles (Figure 8). Overall, vortex circulation and VIA jet magnitudes were directly related over multiple pulsation cycles for a linearly swimming *Eutonina indicans* medusa.

**Figure 9:**
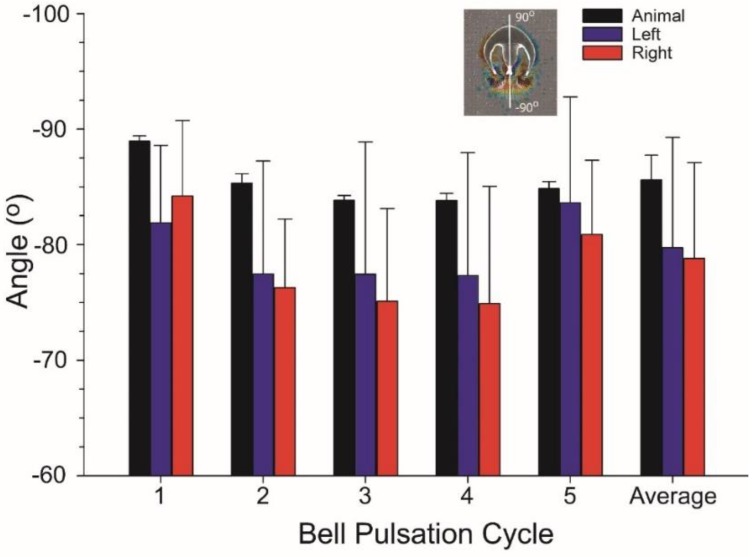
Orientation of animal and VIA axes for an *Eutonina indicans* medusa swimming with a primarily linear orientation. The convention for angular orientation values is illustrated by the inset panel of an animal with a 90, −90° transect line along its central axis. The left (blue) and right (red) values refer to the direction component of the corresponding VIA jets measured during pulsation cycles shown in Figure 2 and Figure 3.

VIA jets were directed opposite and generally parallel to the axis of medusan body motion during straight swimming. VIA jet positions shifted during bell pulsation (Figure 5), resulting in variance of jet directionality. Additionally, differences in starting vortex trajectories on either side of the bell margin (Figure 3) contributed to some variation in VIA jet directionality during bell pulsation (Figure 9). Over complete pulsation cycles, VIA jet directions were not significantly different at either bell margin (ANOVA, Tukeys HSD, *p* = 0.67) but they were lower than the central axis of the animal (ANOVA, Tukeys HSD, *p* < 0.001). The lower VIA angles (Figure 9) directed momentum flux to the right of the animal’s central axis and were accompanied by a small leftward shift in the orientation of the medusa’s central axis (Figure 3 and Figure 9) during swimming.

**Figure 10:**
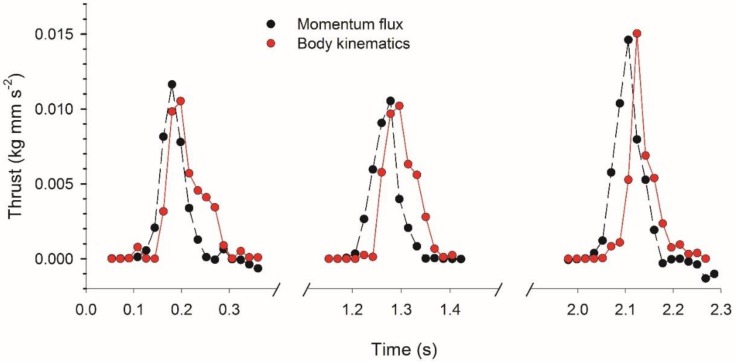
Comparison of thrust estimates based on momentum flux measurements in the near-body wake (e.g., Figure 4) with estimates based solely on body kinematics (subumbrellar volume changes) and fluid jet velocities. The latter approach is similar to that of Daniel (Ref. [4], for estimating thrust by a jet propelled medusa. Note that both methods yield relatively similar results, with a time lag due to temporal differences in body and flow kinematics.

Thrust estimates based on momentum flux measurements were of similar magnitude as those based solely on body kinematics (i.e., a jet model [4]). Peak values for both types of estimates were close during the first three pulsation cycles of *E. indicans* swimming (Figure 10). Although of similar magnitudes, peak values of kinematics-based thrust estimates consistently lagged slightly behind those based on momentum flux. This is potentially attributable to expected temporal discrepancies between body kinematics and vortex ring formation.

## 4. Discussion

### 4.1. Evaluation of Thrust Estimation Methods

The medusan swimming patterns were consistent with momentum flux measurements from the near-body wake of the medusa (e.g., Figure 2C,D). Additionally, if the medusan mass can be represented as a sphere of the observed medusan diameter which is neutrally-buoyant in seawater, then predicted average velocities during bell contraction closely approximated observed velocities (average swimming speeds of 3.9 vs. 4.2 mm s^−1^ for predicted and observed values, respectively, Table 1). The general agreement between the observed and computed patterns of animal motion confirms the expectation that propulsion by an *E. indicans* medusa can be predicted based on analysis of the momentum flux in the symmetry plane of its wake, despite the complex vortex interactions that occur downstream. The similarities between thrust force estimates based on wake momentum flux and those of an alternative, kinematically-based hydrodynamic model (Figure 10) suggest that momentum flux studies can be an effective surrogate in species other than *E. indicans* medusae, where subumbrellar kinematics may not be as accessible.

### 4.2. Mechanical Basis of Jet Thrust Production

One important contribution of this study is the documentation of vortex interactions that provide the fluid mechanical basis of thrust production by *E. indicans* medusae. The model by Daniel [4] used kinematic changes in water parcels ejected from the subumbrellar cavity of a medusa to estimate swimming force production. That model effectively described empirical patterns of swimming by a variety of jet-propelled medusan species [5]. Results reported here are consistent with that model but focus on vortex interactions (e.g., VIAs) involved during fluid jet production rather than estimating changes in subumbrellar fluid volumes during jet production. This focus on the details of fluid interactions complements previous work by explaining how vortices interact to form the jets powering medusan swimming. Maximum jet velocities during contraction produce flows with Reynolds numbers >10^2^ (Re based on jet velocities and velar aperture diameter), indicating that inertial forces strongly influence the wake dynamics and jet formation. DPIV imaging allowed us to link the kinematics of bell motion to generation of rotational flows (Figure 1), jet production (Figure 4), momentum transfer (Figure 5 and Figure 10), and, ultimately, forward body motion (Figure 2B,C). The resulting thrust force estimates are similar to a kinematically-based hydrodynamic model (Figure 10) but, being based on direct flow measurements, provide a more mechanistic understanding of how body-fluid interactions generate thrust. In this way, the results are potentially more generalizable than classical jet propulsion models, which cannot be extended to rowing propulsion [6].

Wake structure patterns demonstrate that momentum transfer from animal to surrounding fluids does not occur evenly through the wake. Instead, momentum in the wake is concentrated at interfaces between opposite-rotation vortices generated by the medusa (Figure 5). While the elevated momentum transfer in the VIAs has been described numerically [17] and visually demonstrated [18], its utility for quantifying thrust to propel and maneuver swimming animals has been largely ignored in the literature. Some exceptions include the model by Ahlborn et al. [19,20] for interactions involving opposite-rotation vortex rings formed by a robotic caudal fin. Ahlborn et al. argued that the rapid dissipation of rotational kinetic energy by interacting opposite-rotation vortices was approximately an order of magnitude more rapid than simple viscous dissipation of the vortices. Hence, the collision of these vortices greatly accelerates energy dissipation from each of the participating vortices. They argued that bending of the fish body generated vortices that transported kinetic energy as rotational momentum, which was then released as linear jets at vortex interfaces by controlled motions of the caudal fin. This energy release depended upon positioning of opposite-rotation vortices for favorable jet thrust production [19,20]. Likewise, Wolfgang et al. [21] found that, through precise body actuation, fish regulate the formation and controlled release of body-generated vorticity, resulting in the production of counter-rotating vortex rings and, hence, a thrust jet. A similar theme has emerged from computational fluid dynamic studies that have documented the alignment of such vortex pairs to create large pressure differences around fish caudal fins that contribute substantially to forward thrust production [22]. Our description of VIA-dominated momentum flux shares common elements with these broader patterns described for swimming fish. The common feature of these different types of swimmers centers on the use of body kinematics to transmit energy into rotation flows within vortex rings. Circulation within these vortex rings is augmented during transport along the swimmer’s body to regions such as the medusan bell margins or fish caudal fins, where the vortex-bound energy is released as linear flows.

Direct measurement of momentum flux away from the swimmer’s body permits evaluation of both thrust force magnitude and direction. Because the VIA jets are strongly linearly directed, their orientation relative to the medusan central axis provides information about the resulting directionality of body motion, as well as its magnitude. For the swimming sequence used within this study, the VIA jet directions at either bell margin were directed slightly away from the medusa’s central axis, generating primarily straight swimming with a small deviation of body motion in the opposite direction of VIA jets (Figure 9). The ability to manipulate VIA jet directionality by vortex positioning plays an important role in fish turning [21] and may also be important for jellyfish maneuvering. In this case, detailed quantitative evaluation of vortex generation and positioning for more specimens than the single one studied here will be essential for developing a rigorous understanding of jellyfish swimming performance. With these detailed mechanisms well quantified, we can leverage advances in soft robotic actuation to create biomimetic vehicles with more efficient jet propulsion than propeller-based analogs [8].

## Figures and Tables

**Table 1:** Comparison of predicted and observed average velocity values during bell pulsation cycles 1–3. The time interval for each cycle was 0.18 s, and the mass of the medusa was estimated as a sphere of the medusa’s bell diameter (1 cm) having a density equivalent to seawater (1.04 g cm^−3^).

Cycle	1	2	3	Average
Peak Force (kg mm s^−2^)	0.0116	0.0105	0.0146	0.0122
Impulse (kg mm s^−1^)	0.0021	0.0019	0.0026	0.0022
Predicted Velocity (mm s^−1^)	3.7	3.3	4.6	3.9
Observed Velocity (mm s^−1^)	3.8	4.1	4.8	4.2
